# Applications of Scheimpflug Imaging in Glaucoma Management: Current and Potential Applications

**DOI:** 10.1155/2016/3062381

**Published:** 2016-12-04

**Authors:** Alexander T. Nguyen, Tiffany Liu, Ji Liu

**Affiliations:** Department of Ophthalmology and Visual Science, Yale School of Medicine, New Haven, CT, USA

## Abstract

Scheimpflug photography is the basis for a variety of imaging devices that are highly versatile. The applications of Scheimpflug imaging are wide in scope, spanning from evaluation of corneal ectasia to quantifying density in nuclear sclerotic cataracts. The potential uses for Scheimpflug-based devices are expanding and a number of them are relevant in glaucoma. In particular, they can provide three-dimensional image reconstruction of the anterior segment which includes assessment of the iridocorneal angle. Photographic analyses allow also for a noncontact method of estimating central corneal thickness (CCT) and intraocular pressure (IOP), as well as the study of various corneal biomechanical properties, which may be useful for stratifying glaucoma risk.

## 1. Introduction

The clinical utility of anterior segment imaging continues to be refined as newer investigations reveal novel applications for their use. As a complement to the slit lamp examination, anterior segment imaging offers qualitative information as well as objective, quantifiable data [1]. Scheimpflug photography is the basis for a number of devices that can image the anterior segment. The technology is highly versatile, with potential applications in the areas of keratorefractive surgery, corneal biomechanics, corneal ectasia evaluation, anterior segment imaging, cataract grading, and surgical planning for femtosecond laser-assisted cataract surgery [2–7].

Herein we review Scheimpflug photography and some of its applications that are relevant to the management of glaucoma patients. Like other anterior segment imaging technologies, Scheimpflug-based devices can provide three-dimensional image representations of the anterior segment, which may be useful for screening narrow angles [1–3]. Intraocular pressure (IOP) is a modifiable and independent risk factor for predicting glaucomatous progression [8]. Its measurement can be affected by corneal parameters like central corneal thickness (CCT). Despite this, there is accruing evidence to suggest that CCT and corneal biomechanical properties are associated with glaucoma independently of their effect on tonometry [6]. The Corvis ST (Corneal Visualization Scheimpflug Technology, Oculus, Wetzlar, Germany) is an ultra-high speed Scheimpflug device that offers a highly reproducible, noncontact method of performing pachymetry, estimating intraocular pressure (IOP), and obtaining corneal biomechanical data [6, 7].

## 2. The Scheimpflug Principle

**Figure 1:**
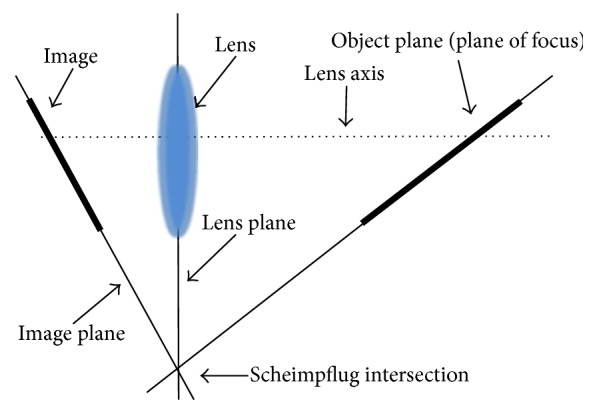
Depiction of the Scheimpflug principle as it applies to photography. When an oblique tangent is extended from the image plane and the lens plane, they intersect at a point that is also intersected by a line extended from the plane of focus. An object that lies on this plane can be captured in focus despite not being parallel with the image plane.

The Scheimpflug principle refers to a concept in geometric optics whereby a photograph of an object plane that is not parallel to the image plane can be rendered maximally focused given certain angular relations among the object plane, the lens, and the image plane (Figure 1) [2]. When applied to ophthalmic imaging, it allows for photographic documentation of the anterior segment with a depth of focus ranging from the anterior cornea to the posterior lens surface [9, 10]. Photographic images of the anterior segment may be variably compressed by the capture of light rays exiting the cornea at unfavorable camera angles. However, this distortion can be minimized by the selection of specific camera angles relative to the slit beam where the capture of reflected light is approximately perpendicular to the corneal surface [1, 11]. This technique is the foundation for the rotational Scheimpflug camera. A select list of Scheimpflug devices is presented in Table 1.

## 3. Anterior Chamber and Iridocorneal Angle Assessment

Recent investigations have explored the emerging role of anterior segment imaging in screening for angle closure [1]. Gonioscopy has served as the diagnostic standard for evaluating narrow angles and related entities [9]. However, this technique requires a contact lens and a proficient examiner to provide a confident diagnosis [3]. And even among experienced clinicians, there is variability in angle grading due to the subjective nature of the assessment. Anterior segment imaging devices hold the potential for a noncontact method of angle closure screening [1, 12, 13]. It may enable practitioners that are less familiar with gonioscopy to effectively screen for angle closure or patients suspected of having angle closure. For experienced clinicians, noncontact imaging may serve as a useful supplement to gonioscopy. It may be particularly valuable in situations where a routine examination is difficult, such as with patients who poorly tolerate gonioscopy or who have difficulty with the required positioning [1, 2, 8]. Several technologies have capabilities that may be useful in the evaluating narrow angles, which are overviewed in Table 2 [1, 3, 12].

The Orbscan (Bausch & Lomb Surgical, Salt Lake City, USA) was commercially introduced in 1995. It is based on a concept referred to as slit-scan triangulation to obtain topographic data. It projects 40 slit beams (20 nasal and 20 temporal) at the anterior segment at an angle of 45° from the axis of the camera [13]. In order for the cornea, the iris, and the lens to be captured in focus, the image plane of the camera is tilted to satisfy the Scheimpflug condition. The measurements obtained by triangulation can then be integrated to provide three-dimensional information regarding the anterior segment. Relevant to the evaluation of angle closure, the Orbscan can estimate the iridocorneal angle and the anterior chamber depth (ACD) [14]. In normal subjects, these measurements have been shown to be highly reproducible [14]. However, studies validating the utility of the Orbscan in assessing angle closure are still needed.

**Figure 2:**
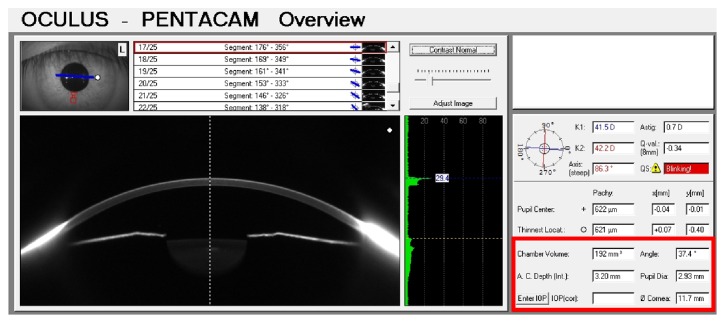
Three-dimensional image representation of the anterior segment obtained by the Pentacam (Oculus, Wetzlar, Germany). Note that visualization of the iridocorneal angle is obscured by total internal reflection. Various parameters obtained by extrapolated measurements may be useful for angle closure screening (red box). These include anterior chamber angle (ACA), anterior chamber depth (ACD), and anterior chamber volume (ACV).

In contrast, the use of the rotational Scheimpflug cameras in evaluating angle closure has been supported with direct comparisons to ultrasound biomicroscopy (UBM) [3] and anterior segment optical coherence tomography (ASOCT) [12]. The Pentacam (Oculus, Wetzlar, Germany) is equipped with two cameras: a rotational camera that captures the Scheimpflug image and a front camera that is used to evaluate the pupillary opening. Information obtained by the front camera aids with measurement corrections as well as the three-dimensional reconstruction (Figure 2) [2]. However, all Scheimpflug-based devices share a similar drawback when compared to UBM and ASOCT. Due to total internal reflection, photographs of the innermost aspects of the iridocorneal angle cannot be obtained and therefore direct visualization of the angle is not possible [1–3]. Scheimpflug devices rely on extrapolated measurements from surrounding structures. On the other hand, ASOCT devices detect the backscatter of reflected infrared light, which allows for high-resolution image reconstruction of the angle, and, to a certain extent, the ciliary body and sulcus [12].

Parameters obtained by the Scheimpflug imaging have been shown to correlate well with gonioscopy [3, 12, 15]. It is capable of estimating the anterior chamber depth (ACD), anterior chamber volume (ACV), and anterior chamber angle (ACA). Kurita et al. [3] compared Pentacam parameters with parallel measurements obtained by UBM (UBM model 840, Humphrey Research Division, Carl Zeiss Inc, Thornwood, NY) in ability to identify patients with narrow angles. The UBM measurement of the ACA was found to have the highest correlation with Shaffer grade [3]; this was consistent with prior works establishing the utility of UBM in evaluating narrow angles [16]. However, the Pentacam's ACA measurement was not reliable for evaluating eyes with a Shaffer grade of 2 or less. The correlation between ACA measurement and gonioscopic grade was also weaker by Schiempflug photography when compared to UBM [17]. The unreliability of the Pentacam's ACA measurement is likely due to limited angle visualization. Nonetheless, the Pentacam's ACD measurement and ACV measurement were shown to be effective at identifying primary angle closure eyes [3, 15]. One study using both UBM and Scheimpflug imaging showed that, in patients with acute angle closure, the fellow eye had findings of narrow ACA width and additional ACA narrowing in response to a light-to-dark luminance change and also pilocarpine-induced pupillary constriction [18].

Rotational Scheimpflug imaging has also been shown to be comparable to ASOCT in partitioning patients with narrow angles. Grewal et al. [12] matched the Pentacam's ACD and ACV measurement against parameters obtained by spectral domain ASOCT. The RTVue 100 (Optovue Inc, Fremont, CA, USA) ASOCT used in their study was equipped with a corneal adaptor module that allows for software to calculate the angle opening distance at 500 microns from the scleral spur (AOD500). The AOD500 is a parameter previously defined using UBM [17]. The Pentacam's estimation of the ACV and ACD outperformed the parameters obtained by the RTVue 100 ASOCT in the detection of narrow angles with gonioscopy as the reference standard [12]. Although the Pentacam cannot directly visualize the angle, it is advantaged by the breadth of three-dimensional data incorporated in its analyses. In contrast, noncontact ASOCT assessment limited to cross sections of only the nasal and temporal angles may exclude representative information regarding the angle. To image the superior and inferior angles, contact would be required to move the eyelids obscuring visualization [9].

Scheimpflug systems such as the Pentacam appear to be viable technologies for evaluating angle closure. Although the Pentacam was better able to predict angle anatomy than the RTVue 100 ASOCT in one study [12], further investigations are needed to discern how rotational Scheimpflug imaging measures up against ASOCT. Presently, both technologies appear comparable in their ability to deliver a noncontact method for screening angle closure. The value of having reproducible, quantifiable parameters is desirable for screening as well as monitoring treatment effect. The Pentacam, for instance, is capable of demonstrating a posttreatment increase in ACV following laser iridotomy [17, 19–23]. However, one clear advantage held by ASOCT and UBM imaging is direct visualization of the angle, ciliary body, and sulcus. This is of particular importance for evaluating mass lesions or conditions such as plateau iris where visualization of ciliary body anatomy is essential to the diagnosis [9, 24].

## 4. Central Corneal Thickness

Goldmann applanation tonometry (GAT) is often regarded as reference standard for measurement of intraocular pressure (IOP). When Goldmann and Schmidt introduced their tonometer in 1957, they acknowledged sources of possible error including CCT. The Imbert-Fick law serves as the basis for GAT. The concept assumes a perfectly thin cornea that behaves like a membrane where the IOP is equal to the applanating pressure. In actuality, the cornea is variably thick rather than perfectly thin, and the tear film contributes a confounding force from surface tension [25, 26].

One of the strongest independent risk factors for developing primary open-angle glaucoma (POAG) is CCT [27–29]. Another independent risk factor is IOP, a measurement which can be influenced by CCT. For this reason, it is believed by some that the CCT is a risk factor for developing POAG only by virtue of its influence on IOP measurement [29]. If this is correct, then we would not expect CCT to be an independent risk factor for developing glaucoma if predictive models corrected for the IOP measurement error attributed to CCT [29]. However, the evidence to date suggests that a thin CCT is associated with an increased risk of POAG beyond its artefactual effect on tonometry [27–30]. For this reason, an interest in corneal properties like CCT will remain relevant regardless of the error it confers towards IOP measurement.

Although ultrasound pachymetry is widely used to measure CCT, it is disadvantaged in several ways [31, 32]. Measurement accuracy and repeatability depend on accurate placement of the probe onto the cornea, which is done manually. Corneal indentation can occur with contact between the cornea and the probe, which may falsely underestimate the actual CCT. And because this technique relies on the assumption that the speed of sound is similar through healthy and diseased corneal tissue, the measurement may be inaccurate in certain pathologic disease states. For these reasons, noncontact methods for estimating CCT are desirable. Devices capable of measuring CCT in this fashion include Scheimpflug imaging devices such as the Pentacam, Galilei (Ziemer, Port, Switzerland), Sirius (Costruzione Strumenti Oftalmici, Florence, Italy), TMS-5 (Tomey, Nagoya, Japan), and the Corvis ST.

Scheimpflug devices are able to provide highly repeatable CCT measurements that are comparable to, but not likely interchangeable with, ultrasound pachymetry CCT [32–36]. Prior studies have shown that highly reproducible CCT measurements can be obtained by the Pentacam, Sirius, Galilei, and Corvis ST. Of these devices, the Galilei has the highest reported intraoperator repeatability. This may be in part attributable to its dual-rotational camera design, which can average the CCT estimate from two different Scheimpflug cameras [32]. However, studies vary widely in reporting how similar CCT measurements are between the different devices. A study comparing CCT measurements obtained by Scheimpflug systems with ultrasound pachymetry has been published previously [32]. Some investigations have noted no difference in mean CCT obtained by either ultrasound pachymetry or with the Pentacam [31, 37]. In contrast, several other studies have noted a significant difference in the mean CCT measured by Pentacam and by ultrasound pachymetry [38–41]. Similarly, the Sirius-CCT measurement is comparable but significantly different from the ultrasound pachymetry CCT [42, 43]. Despite sharing a common imaging technology, the various Scheimpflug devices appear to obtain CCT measurements that are statistically different from each other. Even though these differences may be small, caution is generally advised in comparing CCT values across different measurement platforms. It remains to be clarified whether these differences may be sufficiently small for them to be negligible in clinical contexts.

## 5. Corneal Biomechanics and IOP

CCT is but one dimension of a multifaceted area of study that comprises corneal biomechanics. Interest in the biomechanical properties of the cornea parallels our interest in CCT: it may help explain the source of measurement error in tonometry and how structural features of the cornea can predict the risk of glaucomatous progression independent of their effect on IOP. However, the mechanisms underpinning how CCT and corneal properties confer a risk towards developing glaucoma remain unclear.

Theoretical models predict that optic nerve head biomechanics can be influenced by the structure of the adjacent sclera [44, 45]. The optic nerve head is a site of discontinuity within the cornealscleral shell [45] and is the location of retinal ganglion cell injury due to glaucoma [45]. From theoretical models, the lamina cribrosa is predicted to experience an increase in tensile strain with increasing eye radius [44, 46]. These models also predict, though, that scleral stiffness may mitigate this increase in strain expected from having a longer eye [44, 46]. It has been hypothesized that corneal parameters like CCT may serve as surrogate measures for some biomechanical property of the sclera [44, 46]. Lanzagorta-Aresti et al. [47] showed that changes in the displacement of the lamina cribrosa after medical treatment of IOP was significantly correlated corneal hysteresis. Furthermore, Wells et al. [48] demonstrated that low corneal hysteresis, but not CCT, was associated with reduced optic nerve head compliance. However, efforts to correlate CCT with lamina cribrosa thickness have yielded no significant association between the two [49].

The two devices capable of quantifying biomechanical features of the cornea include the Ocular Response Analyzer or ORA (Richert Ophthalmic Instruments, Depew, New York, USA) and the Scheimpflug-based noncontact tonometer Corvis ST [7, 50]. To date, most of the studies investigating corneal biomechanics have utilized the ORA, which was released in 2005; the Corvis ST was made available in 2012. The ORA can measure corneal hysteresis, which may be an indicator of the cornea's viscoelasticity [51]. Patients with POAG and normal tension glaucoma have been shown to have lower-than-average corneal hysteresis values [52]. Furthermore, low corneal hysteresis has also been implicated in glaucomatous field progression [53, 54].

**Figure 3:**
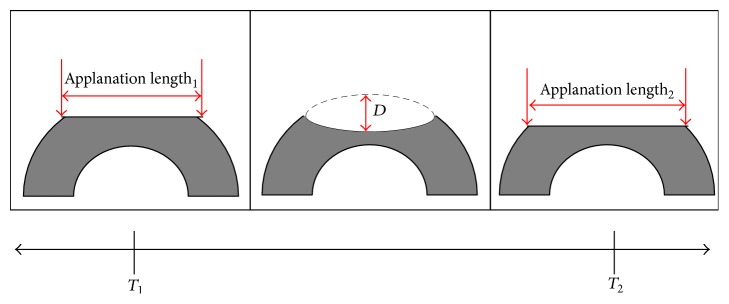
Diagramatic representation of the biomechanical response of the cornea to the metered air puff delivered by the Cornea Visual Scheimpflug Technology (Corvis ST). The first phase (left) is marked by corneal surface flattening and the initiation of an inward conformational change in the corneal curvature (referred to as the first applanation *T*_1_). Further deformation produces a concave corneal surface. The moment it reaches the maximally deformed state (middle) is referred to as the time of highest concavity. The distance, *D*, is the peak distance or corneal deformation amplitude. After reaching its maximally concave shape, the cornea recoils into its original shape. When the surface is similarly flattened compared to *T*_1_, this moment marks the second applanation or *T*_2_ (right).

The Corvis ST provides a noncontact method for evaluating IOP, CCT, and the cornea's biomechanical response to a collimated puff of air. It is equipped with an ultrahigh-speed Scheimpflug camera that is capable of recording 4330 frames/second. The air puff is delivered with a fixed pressure at the corneal surface over 31 ms, allowing for the digital capture of 140 images. The corneal response to the air puff is initially marked by an inward conformational change in the corneal curvature. This initial flattening of the cornea is referred to as the first applanation (Figure 3) [50, 55]. The cornea eventually deforms to a point where it is maximally concave prior to returning to its original shape. This event defines the corneal deformation amplitude at the highest concavity (Figure 3). The cornea then naturally returns to its original shape, which is referred to as the second applanation [50, 55]. Various aspects of the corneal deformation response to the air-puff can be quantified, which are reviewed in detail elsewhere [55].

Recently, Lee et al. [50] published a cross-sectional study demonstrating a parameter obtained by the Corvis ST that appeared to be independently associated with glaucoma risk. They identified three Corvis ST parameters that could partition patients with either POAG or normal tension glaucoma from normal controls: (1) outward applanation velocity, (2) time to highest concavity, and (3) peak distance. However, two of these parameters, namely, outward applanation velocity and peak distance, were associated with Corvis-IOP [50]. On the other hand, the highest concavity time was associated with the glaucomatous group and not dependent on other established risk factors for glaucoma such as CCT or IOP. It should be noted, though, that their results contrast with those of Leung et al. [7] in that the latter study group did not find any of the Corvis ST parameters to be capable of partitioning glaucomatous patients from normal subjects.

While various corneal parameters may be associated with glaucomatous risk independently of their effect on IOP measurement, the study of corneal biomechanics may aid in better understanding sources of measurement error. IOP is a modifiable risk factor associated with glaucomatous progression [27–30]. A coveted goal in glaucoma management is approximating IOP, as close as possible, to the “true” IOP. An intracameral measurement of IOP may be the most accurate method for achieving this end, but it is clinically impractical [56]. Because of this, various approaches have been developed and described to help correct for the effects that biomechanical features of the cornea have on IOP measurement. Regression analyses have been proposed with formulaic corrections for values obtained by applanation tonometry, taking into account the effects of CCT. There is growing evidence, though, that corneal biomechanical properties, such as corneal hysteresis, may better explain the source of measurement error in tonometry than CCT [56].

The corneal deformation amplitude measured by the Corvis ST has been implicated as source of measurement error of GAT. In a study population containing normal and glaucomatous eyes, Leung et al. [7] compared IOP measurements obtained by GAT with dynamic contour tonometry (DCT, Pascal, Swiss Microtechnology AG, Port, Switzerland). IOP measurement by DCT is theoretically less dependent on corneal biomechanical properties and by this virtue it may better approximate the “true” IOP. Indeed, IOP measurements obtained by DCT are highly concordant with intracameral measurements [57]. To evaluate factors that may explain the difference in IOP measurements obtained by DCT and GAT, Leung et al. [7] also studied the biomechanical properties obtained by the ORA and the Corvis ST. Measurement of the corneal deformation amplitude was shown to be dependent on IOP and CCT (measured by ultrasound pachymetry). From their univariate analysis, the difference in DCT and GAT measurements was associated with CCT and corneal deformation amplitude after adjusting for the effect of IOP. But with the multivariate analysis, the only parameter significantly associated with this measurement difference was corneal deformation amplitude. The authors comment that the influence of CCT on GAT likely stems from the effect of CCT on corneal deformation amplitude [7]. Of note, the corneal hysteresis and corneal resistance factor measurements obtained by the ORA were not significantly associated with the measurement discrepancy between DCT and GAT in this investigation [7]. The ORA functions in a very different way from the Corvis ST; while the air puff of the Corvis ST is applied with a fixed force, the ORA air puff is delivered with a variable force. Although both instruments assess corneal biomechanics, it is difficult to compare the metrics obtained by these two devices [55, 58].

Although the Corvis ST is capable of providing an estimate of IOP, Leung et al. [7] did not compare the accuracy of this measurement with DCT. The IOP estimate by the Corvis ST has been shown to be highly reproducible [59] and comparable to GAT [60, 61]. It should be noted, though, that the degree of correlation between Corvis-IOP [59] and GAT was relatively weak compared to other techniques such as DCT [62] and iCare rebound tonometry [62, 63]. The significance of this finding is unclear absent data validating the accuracy of Corvis-IOP measurements [59, 61]. Future investigations comparing intracameral IOP measurements to the Corvis ST would be helpful for this end [60]. There is no consensus agreement among studies on whether the Corvis-IOP tends to be higher [60, 63] or lower [61, 64] than the IOP estimated by GAT.

## 6. Cataract Evaluation

A brief section on cataract evaluation is reviewed given its relevance in glaucoma management and especially in light of the rising interest in combining cataract extraction with minimally invasive glaucoma surgery (MIGS). MIGS procedures offer the potential for modest reductions in IOP in patients with mild-moderate glaucoma. They are not always performed as standalone procedures given that their IOP-lowering effect cannot rival that of traditional filtering surgeries. Because of this, it is often preferable for MIGS procedures to be done conveniently at the time of cataract surgery [65].

Traditionally, the appraisal of a visually significant cataract involves two main factors: (1) a functional deficit, such as Snellen acuity, and (2) the clinician's assessment of lens opacification. There are shortcomings associated with both arms of the traditional approach. Patients with relatively good Snellen acuity may actually have poor vision quality and the subjective grading of lens opacification is subject to interrater variability. The most widely recognized lens-grading schema is the Lens Opacification Classification III (LOCS III), which was last updated in 1993 [66].

Scheimpflug systems such as the Pentacam may be able to enhance our assessment of cataractogenesis. The Pentacam can measure lens densitometry with specific metrics that include average density and maximum density. Based on these measurements, the Pentacam can be equipped with software that then assigns a grade of nuclear sclerosis on a scale of 1–5 (Pentacam Nuclear Staging or PNS). The PNS score has been shown to correlate well with Snellen acuity and LOCS III grade, validating the Pentacam's automated assessment of nuclear sclerosis [67]. Furthermore, the Pentacam's densitometric parameters have been positively correlated with higher-order aberrations (HOAs) obtained from wavefront analyses [67]. A consideration of HOAs may enable clinicians to better appreciate why patients with relatively good Snellen acuity may complain of poor vision quality. Similar such innovations are likely to increase our sensitivity in identifying visually significant lens opacities, thereby potentially expanding our indications for cataract extraction.

The use of Scheimpflug systems as surgical planning tools has also been suggested. Measurements of lens densitometry enable a quantitative evaluation of a nuclear sclerosis; this may help guide the selection of phacoemulsification technique or use of a femtosecond laser for lens fragmentation. It should be mentioned, however, that lens densitometry measurements are currently less precise for higher-grade nuclear cataracts [67]. Scheimpflug imaging has been shown to be helpful for evaluating intraocular lens tilt and decentration following cataract extraction [68, 69]. The LENSAR (LENSAR Inc., Winter Park, USA) is a femtosecond laser that is equipped with Scheimpflug imaging capabilities [70]. Similar to the Pentacam, the device can automatically grade lens density on a scale of 1–5. It special features an imaging system that enables the detection of any tilt that may be exhibited by the native crystalline lens; this is important for maximizing the likelihood of a producing a precise, free-floating capsulotomy [70].

## 7. Summary

The Scheimpflug principle is the basis for a number of devices and imaging systems. The technology is extraordinarily versatile, with applications spanning from laser keratorefractive surgery to quantifying cataractogenesis. Scheimpflug devices have several relevant applications for glaucoma management. Currently, Scheimpflug-based imaging systems have formidable capabilities to ASOCT for predicting angle closure, despite their inability to visualize the iridocorneal angle. Noncontact methods of assessment have multiple advantages including sanitary considerations, patient comfort, and in some cases, less operator-dependent. Because of this, technologies like Scheimpflug-based devices are likely to be increasingly used as they become more accessible. The clinician should heed caution, though, in interchanging measurements obtained by different technologies as they can be slightly different. With the introduction of the Corvis ST, investigators have 2 available devices for the study of corneal biomechanics. Relevant investigations with these devices are needed to provide unanswered questions for how corneal parameters can be related to glaucoma beyond their impact on IOP.

## Figures and Tables

**Table 1:** Select Scheimpflug imaging systems.

Device	Manufacturer	Image acquisition
Orbscan II	Bausch & Lomb, USA	Horizontal cross section
Pentacam	Oculus, Germany	Single rotating camera
Galilei	Ziemer, Switzerland	Dual rotational camera
Sirius	CSO, Italy	Single rotating camera
TMS-5	Tomey, Japan	Single rotating camera
Precisio	Ivis, Italy	Single rotating camera

**Table 2:** Comparison of anterior segment imaging modalities for assessing narrow angles.

Imaging system	Correlation with gonioscopy	Quantitative parameters	Advantages	Limitations
Slit scan topography	N/A	Iridocorneal angle ACD	Noncontact	No visualization of angle, ciliary body or sulcus

Rotational Scheimpflug camera	++^*∗*^	ACD ACV ACA	Noncontact	No visualization of angle, ciliary body or sulcus

ASOCT	+++	AOD500 TISA500	Noncontact Direct angle visualization Some visualization of ciliary body and sulcus	Requires identification of scleral spur Noncontact assessment limited to temporal and nasal angles

UBM	+++	ACD ACV ACA AOD500 TISA500	Excellent visualization of angle, ciliary body and sulcus	Requires contact, identification of scleral spur

ACD: anterior chamber depth, ACV: anterior chamber volume, ASOCT: anterior segment OCT, UBM: ultrasound biomicroscopy, AOD: angle opening distance, and TISA: trabecular iris area.

N/A: not available, validating studies required.

*∗* indicates that it may be as useful as ASOCT for partitioning narrow angles but it does not provide direct angle visualization.
